# Midbrain hematoma presenting with isolated bilateral palsy of the third cranial nerve in a Moroccan man: a case report

**DOI:** 10.1186/1752-1947-6-197

**Published:** 2012-07-16

**Authors:** Ouarda El Ouali, Ouafae Messouak, Mohamed Faouzi Belahsen

**Affiliations:** 1 Department of Neurology, University Hospital Hassan II, Fez, Morocco

## Abstract

**Introduction:**

Bilateral third nerve palsy secondary to a hemorrhagic stroke is exceptional. To the best of our knowledge, no similar case has been reported in the literature.

**Case presentation:**

We describe the case of a 69-year-old Moroccan man who presented with isolated sudden bilateral third nerve palsy. Computed tomography (CT) of the brain revealed a midbrain hematoma. The oculomotor function gradually and completely improved over eight months of follow-up.

**Conclusion:**

Stroke should be included in the differential diagnosis of sudden isolated oculomotor paralysis even when it is bilateral because of the severity of the underlying disease and the importance of its therapeutic implications.

## Introduction

Cases of stroke that result in isolated third nerve palsy without other significant non-ocular signs are rare. Besides a few sporadic cases, no studies including a sufficient number of cases and describing a clinical-radiologic correlation have been reported [1]. Isolated third nerve palsy secondary to a hemorrhagic stroke is rare [2]. Isolated bilateral third nerve palsy secondary to a hemorrhagic stroke is exceptional. To the best of our knowledge, no similar case has already been reported in the literature. A case of bilateral trochlear palsy following a brainstem hematoma has been described [3]. We report the case of isolated bilateral third nerve palsy secondary to a midbrain hematoma.

## Case presentation

**Figure 1:**
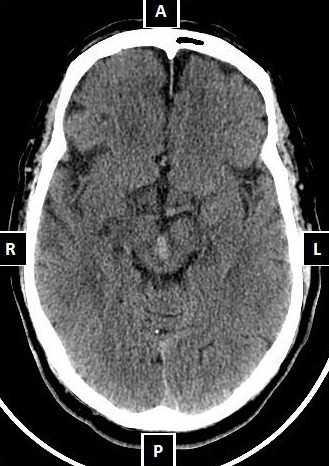
Cerebral computed tomography (CT) scan showing a spontaneous hyperdensity lesion of the midbrain consistent with a midbrain hematoma.

A 69-year-old Moroccan man, with a medical history of untreated hypertension, developed suddenly diplopia, a droop of both upper eyelids with the inability to open his eyes. The symptoms were constant and did not fluctuate during the day. He reported a hypertensive episode prior to these symptoms. He denied any weakness, numbness or change in mental status. Initial physical examination found bilateral ptosis with a limitation in adduction, elevation and depression of both eyes which was more marked on the right side. Both pupils were in miosis. The remaining neurological examination was strictly unremarkable. His blood pressure was 220/120mmHg on admission. Initial blood tests showed a normal full blood count, normal urea, electrolytes and C-reactive protein. Computed tomography (CT) of the brain revealed a spontaneously high-density lesion of the midbrain consistent with a midbrain hematoma [Figure 1]. The oculomotor function gradually and completely improved over eight months of follow-up.

## Discussion

The third cranial nerve innervates the levator palpebrae superioris and most ocular muscles (except the lateral rectus and superior oblique). With its parasympathetic fibers, it innervates the iris constrictor and the annular portion of the ciliary muscle. The nuclei of the third cranial nerve originate at the level of the superior colliculus.

The oculomotor fascicles sweep ventrally and laterally from the oculomotor complex, pass through and medial to the red nucleus and exit the brainstem medial to the cerebral peduncles [2].

Thus in the midbrain, a third nerve palsy results either from nuclear involvement or from the involvement of the oculomotor fascicles on their way to the interpeduncular sulcus.

Oculomotor palsy is usually associated with various neurological syndromes related to very specific diseases. It may be observed in many etiological circumstances: infectious, vascular, traumatic, toxic and metabolic disorders. Stroke rarely causes isolated oculomotor palsy.

In theory, ischemic stroke cannot result in bilateral involvement of the third nerve since the midbrain arterial supply arises from vessels emerging from both sides. However, a bilateral involvement is conceivable when both sides are supplied with a single arterial trunk. In fact, midbrain infarcts may result after occlusion of the artery of Percheron and they usually affect the periaqueductal gray matter where the third cranial nerve nuclei are located. In this context, a bilateral oculomotor palsy could likely occur but has not yet been described. Unilateral third nerve palsy was reported in a bilateral thalamic stroke involving the rostral midbrain due to the occlusion of the artery of Percheron [4].

To the best of our knowledge, no case of midbrain hemorrhagic stroke leading to bilateral third nerve palsy has been described in the literature.

In our patient, the bilateral third nerve palsy can be explained by both nuclear and/or fascicular involvement.

The sudden onset, preceded by high blood pressure (220/120mmHg), is suggestive of hemorrhagic midbrain stroke.

The absence of an associated impairment of the descending and ascending tracts suggest a posterior bilateral midbrain lesion.

Computed tomography (CT) of the brain, showed a spontaneously hyperdense area in the tectum immediately ventral to the aqueduct of Sylvius and extending to the interpeduncular sulcus (Figure 1).

Our patient had complete bilateral extrinsic third nerve palsy, associated with miosis. Usually, when the paralysis of the third nerve affects intrinsic motility, it is a paralytic mydriasis with abolition of pupillary reflexes. In light of the speculation that a lesion along the dorsal region of the Edinger-Westphal nucleus damages the pretectal fibers of pupillary light reflex, the miosis observed in our patient could be explained by the interruption of the descending supranuclear inhibitory fibers to the Edinger-Westphal nucleus. Devoid of its central inhibition, this nucleus would continuously release parasympathetic impulses to the iris sphincter resulting in a tight miosis [5].

The true incidence of isolated spontaneous midbrain hemorrhage is difficult to determine. Its frequency has been variously linked to the number of intracerebral hemorrhages, posterior fossa hemorrhages and brainstem hemorrhages. Thus, comparison of the reports is difficult or impossible. Among the 14 locations in the brainstem of Boudouresque’s series of 318 patients, only five were located in the midbrain. In the series of Reason et al., among the 25 brainstem spontaneous hematoma that were recorded between 1990 and 2000, 22 were pontine and only three were peduncular [6]. In hypertensive patients, the most common site of brainstem hematoma is the pons. Midbrain localization is much rarer. There were no cases of midbrain hemorrhage in Freytags anatomopathologic series of 393 patients who died from hypertensive intraparenchymal hemorrhage [6].

Data gained from clinical pathology firmly establishes that the paramedian tegmental zone immediately ventral to the ventricular system in the midbrain and pontine area is critical to consciousness in humans [7]. No disturbance of consciousness, even transient, was observed in our patient.

Midbrain hematomas are characterized by high mortality; however, there are a few forms compatible with a survival of sufficient quality [8,9]. The prognosis of brainstem hematomas depends mainly on the hematoma size. In the series of Komyama, severe hematomas had a size that exceeded 25mm. In the series of Sanon, all hematomas exceeding 11mm resulted in death [8]. In our patient, the hematoma measured 11.1mm and there has been a marked improvement in his ocular disorders after eight months of follow-up.

The paucity of symptoms, the absence of hydrocephalus due to aqueductal compression and the relatively fast recovery in the absence of surgery are unusual for a midbrain hemorrhage [10].

## Conclusions

Isolated bilateral third nerve palsy can occur secondary to a midbrain hematoma. This is an exceptional situation that must be considered because of the severity and therapeutic implications of the underlying disease. Stroke should be included in the differential diagnosis of sudden isolated oculomotor paralysis even when it is bilateral. The description of other cases with good clinical and radiological material will certainly improve our current understanding of oculomotor disorders of vascular origin.

## Consent

Written informed consent was obtained from the patient for publication of this case report and any accompanying images. A copy of the written consent is available for review by the Editor-in-Chief of this journal.

## Competing interests

The authors declare that they have no competing interests.

## Authors’ contributions

OM examined the patient and analyzed and interpreted investigative data. MFB and OE examined the patient and analyzed and interpreted investigative data, performed a literature review and contributed in writing the manuscript. All authors read and approved the final manuscript.

## References

[B1] Kim JS Kang JK Lee SA Lee MC Isolated or predominant ocular motor nerve palsy as a manifestation of brain stem stroke Stroke 1993 24 581 586 10.1161/01.STR.24.4.581 8465365

[B2] Shintani S Tsuruoka S Minato Y Shiigai T Radiologic-clinical correlation, isolated third nerve palsy caused by midbrain hemorrhage Am J Neuroradiol 1994 15 1508 1511 7985571 PMC8334411

[B3] Tachibana H Mimura O Shiomi M Oono T Bilateral trochlear nerve palsies from a brainstem hematoma Clin Neuroophtalmol 1990 10 35 37 2139047

[B4] López-Serna R González-Carmona P López-Martínez M Bilateral thalamic stroke due to occlusion of the artery of Percheron in a patient with patent foramen ovale: a case report JMedCase Rep 2009 3 7392 10.4076/1752-1947-3-7392 PMC2767135 19918273

[B5] Miller NR, Newman NJ Walsh and Hoyt’s Clinical Neuro-Ophtalmology The Autonomic Nervous System: disorders of pupillary function, accommodation and lacrimation. Volume 1 2005 Sixth edition Lippincott Williams and Wilkins, 739 804

[B6] Michael J Link JD Bartleson D Glenn F Frederic B Meyer B Spontaneous midbrain hemorrhage: report of seven new cases Surg Neurol 1993 39 58 65 10.1016/0090-3019(93)90112-E 8451723

[B7] Tomecek FJ Morgan JK Ophtalmoplegia with bilateral ptosis secondary to midbrain hemorrhage. A case with clinical and radiologic correlation Surg Neurol 1994 41 131 136 10.1016/0090-3019(94)90110-4 8115950

[B8] Thiam A Ndiaye M Hématome pédonculaire bénin: A propos de deux cas révélés par la tomodensitométrie Méd Afr Noire 2000 47 520 52422993718

[B9] Abrar A Anil D Isolated third nerve palsy due to mesencephalic hematoma Indian J Neurotrauma 2007 4 53 54

[B10] Shuaib A Murphy W Mesencephalic hemorrhage and third nerve palsy J Computed Tomography 1987 11 385 389 10.1016/0149-936X(87)90078-6 3443013

